# Address of the Royal Somerset Jennerian Society, to the City of Bath and County of Somerset

**Published:** 1805-10-01

**Authors:** 


### Address of the Royal Somerset Jennerian Society, to the City of Bath and County of Somerset.

THE Committee of this Society, deeply lamenting the extensive ravages lately occasioned and still continued by the prevalence of Small-Pox in this City, beg leave to call the public attention to some deplorable facts, and to the causes which continue to obstruct the general adoption of Vaccine Inoculation, which is demonstrated, by the experience of nearly the whole civilized regions of the world, to possess the power of annihilating this dreadful distemper.

This Committee have taken pains to investigate the actual number of deaths from small-pox in this City, from the 1st of May last to the present period; and although the numbers below are lamentably great, yet they are probably inferior to the actual amount, many burials having taken place in other grounds than those belonging to the respective parishes. In the course of their inquiries, the Committee have met with numerous instances of the vaccinated remaining secure amidst the strongest influence of small-pox contagion.

Died of small-pox, from May 1st, 1805, to July 22d, in the following parishes:

| | |
|---|---|
| Walcot | 15 |
| St. Peter and Paul | 8 |
| St. James } Lyncombe and Widcombe } | 18 |
| St. Michael's | 3 |
| Bathwick | 16 |
| In all | 60* |

---

* The fixed population of Bath is 34,000 persons.

In a sketch of a plan to exterminate the small-pox, published by Dr. Haygarth in 1793, it was proved by authentic facts (page 31) that "In London the births are to the deaths by small-pox at 6¼ to 1, in Manchester at 6½ to 1, in Liverpool at 5½ to 1, in Chester at 6 2-3d to 1. If from the number born we subtract all who have been inoculated, and all who die of other diseases before they are exposed to the casual contagion, the intelligent reader will be convinced that the casual small-pox proves fatal to a *full fifth part of the patients* who are attacked by this dreadful pestilence.

The proportional mortality of the casual small-pox is probably not less in Bath and all other large towns. It falls almost entirely upon poor children; for all the higher and middle classes are inoculated.

The Committee are convinced that it cannot be necessary for them to enlarge on the nature and extent of this devastation, or to point out its melancholy tendency to the sense and humanity of the public.

The mortality from small-pox in this city since the 1st of May last, is full three-fourths of the average of that in London and its environs from the same disease; where, for the last two years, it has been reduced more than three-fourths of the average of the past 50 years, by the practice of vaccine inoculation; particularly since the foundation of the Royal Jennerian Society, as will be seen by the following extract from the Quarterly Report of that Society:

"June 5, 1805.—We have the satisfaction to observe, that the deaths by small-pox within the bills of mortality appear still to be considerably reduced. In five months, ending with May 1804, the deaths were 359; and in the same period, 1805, they are only 147—making a diminution of 212 deaths. This is certainly a subject of congratulation; but it is yet a matter of serious regret, that so many valuable lives are still lost, when the means of total prevention are in our power. We are incited to bring this subject under the consideration of the Quarterly Court, in consequence of authentic information received, that in several of the most populous cities in Europe the small-pox appears to be annihilated, by vaccine inoculation having been adopted with a zeal and energy far superior to what has yet been manifested in this country, where the discovery originated."

With these incontestible facts within their knowledge, contrasted with the distressing devastation just described, and and with their information of similar destruction going on in various other places from the same cause, this Committee consider it as the bounden duty of the friends of mankind to support, diffuse, and encourage the practice of vaccine inoculation; and they perceive with regret, that at a period when the vigorous co-operation of the philanthropic and well-informed is called on for this purpose, endeavours are made to diminish the public confidence in, and to depreciate the reputation of, this most valuable and best preventive of the evils enumerated.

In respect to the imputed failures of vaccine inoculation to prevent small-pox, this Committee conceive that they cannot generally reply to them better than by adducing an extract from the Quarterly Report of the Royal Jennerian Society, June 5, 1805; also from the Resolutions of the Vaccine-Pock Institution:—

"We are fully persuaded that greater importance has been attached to the cases of supposed failures than they deserved, as, on investigation, most of those cases have been ascertained to arise from some irregularity in practice, or some other assignable cause."

### *Resolutions of the Vaccine-Pock Institution.*

"1st. That it appears from the numerous reports that have been transmitted or attested by the members of the medical establishment of this Institution from abroad, from our own country, and from their own experience, that the proportion of failures in the cow-pox inoculation to give security against the small-pox which have been published, does not amount to more than 50 out of 250,000 vaccinated persons.

"2d. That it does not appear on examination of the published reports of these failures, and upon the investigation of many of them by the medical establishment of this Institution, that *ten* have been substantiated by admissible evidence."

It appears also from a Report lately published by the Cow-Pock Institution in Dublin, "that not a case has occurred to excite the smallest doubt as to the permanent efficacy of cow-pock in protecting the system against small-pox, in Dublin, or its neighbourhood."—The reports from Paris, Vienna, Berlin, Geneva, America, and the East and West Indies, in all which countries and cities vaccine Inoculation has been extensively diffused, whilst they exhibit the pleasing picture of the diminution of small-pox in some situations, and its absolute extinction in others, no where even mention exceptions to its efficacy.

 The

The Committee hope and believe, that these general facts would be adequate to the conviction of every intelligent and impartial mind; but they are induced to pay more particular attention to two cases in Bath, which have been lately promulgated, and have been erroneously asserted to be instances of the failure of vaccine inoculation.

One of these cases was published in the Bath Herald, and the Bath Journal of the 21st and 24th of June last; and contained an assertion that Elizabeth Grace, of Grove-street, then dead of small-pox, had been vaccinated some years ago by Mr. Barnes, surgeon, of Pewsey. This Committee became desirous of the elucidation of the fact, and Dr. Parry, one of its members, addressed Mr. Barnes for information on the subject, from whom he received the following reply:

"Dear Sir, *Pewsey, July* 2, 1805.

" In reply to your favour respecting a paragraph inserted in the Bath Journal of the 24th of June, stating that I had about four years since inoculated with cow-pox Mrs. Elizabeth Grace and her brother, I beg to say that it had no foundation in truth. Dr. Parry has favoured me with a letter on the same subject; and I have made enquiries of the parents of this young woman, who are residents in this parish: they say the operator on this occasion was a journeyman shoe-maker, by name Charles Pearce, who I know vaccinated many people in this place and neighbourhood about that time. This man is since dead, and I cannot learn from whence he procured the matter.

" I have written to the printer of the Bath Journal, requesting to know on what authority he inserted the paragraph.

" You are at liberty, Sir, to make what use you please of this letter, and I remain your obedient servant,

*Dr. Parry, Bath.* J. Barnes."

Another case, that of Peter Coday, in Lansdown-road, has been asserted, in a pamphlet lately published for the express purpose of attempting the discredit of vaccine inoculation, to have been an instance of its failure. Information of this supposed failure having been given to the Committee, the child in question was examined, together with its mother, on July 15th ult. by Doctors Parry, Haygarth, and Crauford, and by Mr. Tudor and

Mr.

Mr. Creaser, Members of the Committee. These gentlemen, from the existing appearances, and the history of the case as detailed by the mother, together with the account furnished by H. White, Esq. and Mr. Roe, apothecary, *(who was positively asserted by the mother to have been the only medical gentleman who saw the child during the eruption)* unanimously declare their entire disbelief that Peter Coday's disease was small-pox.

Such this Committee firmly believe to be the general description of facts supposed to be adverse to vaccine inoculation; and whilst they lament their influence amongst the ignorant and inferior classes, they cannot too strongly express their reprehension of the publication of such occurrences, on grounds frivolous, imperfect and even false. To judge correctly of such instances, and of the comparative value of vaccine inoculation, requires information, judgement, and extensive opportunities of observation. The practitioners of Medicine and Surgery, both in this and other countries, most distinguished in these respects, have been and still continue to be, the most ardent favorers of the practice.

This Committee do no pretend to affirm, that in the present state of our knowledge, instances of exception to the general security produced by vaccination do not occur. Such are well authenticated to have succeeded to small-pox inoculation in various instances which have fallen under the observation of the Members of this Committee. But such cases, were they even in a larger proportion than the present supposed failures bear to the aggregate of vaccine inoculations, would, on the most obvious calculation, place the cow-pox in the highest light of relative estimation, and fully entitle it to rank as the most fortunate of discoveries for the preservation of the human race.

This Committee do further beg leave to call the public attention to the mischiefs attendant on small-pox inoculation, a practice certainly *not morally justifiable* under the present circumstances, as it is a constant source of the diffusion of the natural disease so extensively fatal. It is a fact, proved by the most authentic documents, that partial small-pox inoculation has increased the whole mortality from small-pox for a long series of years succeeding to its introduction; and that its preservation and propagation are now no more to be vindicated than that of any other mischievous contagion. Vaccine Inoculation has already begun to diminish this mortality; and if adequately supported by the influence and arguments of the betterbetter-informed and liberal directed, to the conviction of the ignorant and prejudiced, its general adoption must ultimately abolish this dreadful pestilence.

